# From frustration to enlightenment: experiences of student exchange program awardees in Taiwan

**DOI:** 10.12688/f1000research.51865.2

**Published:** 2021-11-29

**Authors:** Wolter Parlindungan Silalahi, Friska Ria Sitorus

**Affiliations:** 1 Department of Tourism, Institut Agama Kristen Negeri Tarutung, Tarutung, North Sumatera, 22411, Indonesia; 2 Department of Education and Human Potentials Development, National Dong Hwa University, Hualien, Hualien, 974301, Taiwan

**Keywords:** Experience, Qualitative Study, Student Exchange

## Abstract

**Background: **This qualitative study investigates the experiences of international students’ exchange who faced difficulties in adjusting to their new environment. They experienced awkwardness in the use of advanced equipment from technology and in various cultural aspects. This study investigates two Indonesian students who participated for a semester at a Taiwanese university. It aims to explore the Indonesian student exchange program awardees’ experiences by examining both the obstacles and benefits of attending the Taiwanese university.

**Methods: **This study used qualitative data analysis. Data were collected through a semi-structured interview, informal participant observations, and a set of open and closed-ended questions. Two Indonesian undergraduate students who belonged to the same major, year, and university were recruited to participate in this study.

**Results: **Challenges and Benefits are the two major themes in this research. Challenges include: (1) Departure and journey; (2) Difficulties in settling in; (3) Inability to use technology facilities. Benefits include: (1) Meeting new people and exploring new places; and (2) 21 ^st^-century classroom environments.

**Conclusions:** Despite the challenges that are associated with being students exchange awardee, there are vast opportunities for self-development and learning that is associated with teaching from the 21st-century classroom pedagogy.

## Introduction

The globalization era has led to a significant increase in students' exchange programs abroad and exchange programs can contribute to diversity and internationalization of classrooms, colleges, and communities and to the enhancement of mutual understanding and appreciation of the differences around the world. Cheney (2001) stated that international interaction could generate two specific benefits, namely mastering of English or other foreign language and increasing international friendships. In addition, discussions with international students make significant, positive effects on critical thinking and promote friendship.

Recently, many universities develop peer pairing programs to match those running students exchange programs with local students ( Summers et al., 2008). This is one of the ways to expand university relations internationally, to improve the quality of a university, and to increase student activities internationally. It aims to improve their education areas in ways that might be useful in future life. Education has a lot of interesting opportunities for humans that can give us better chances to obtain good careers and well-paid jobs. These opportunities can help to build peace, prosperity, harmony, happiness, stability, self-esteem, and wisdom in individuals. The student exchange program also provides benefits to universities and students by improving the quality of education. Studying abroad also provides opportunities to learn about other cultures. Many students aim to take part in these program so that they can learn how to appreciate and respect other people's culture and see it as a reflection of their own ( Lai, 2018). In addition, studying abroad can increase their personal growth, awareness, self-efficacy ( Edmonds, 2010). They also learn how to socialize in a community, immerse themselves in a new language, make new friends with local or international students, and gain experience from being international students. Being part of student exchange is a priceless experience that provides many advantages such as enhanced intercultural learning, global awareness, self-development, language acquisition, and many other positive long-term impacts ( Giedt et al., 2015). In sum, the exchange student program creates opportunities to learn, work, gain insight, and to become a problem solver in life.

On the other hand, there are also many challenges, such as homesickness, lack of adequate finance, adaptation to new problems, prejudices, and housing and transportation related problems ( Crockett & Hays, 2011). In addition, there are vocational, physical health, social, and academic issues can occur ( Yi et al., 2003). Other adjustments related to academic areas include learning style, study habits, educational background, culture and language proficiency ( Andrade, 2006). The new environment also requires social issues including the lack of understanding of local context ( Park, 2010), and differences in punctuality ( Razack, 2002).

Based on a study conducted by the International Educational Consultants Association, there were approximately 35,000 Indonesian students who studied abroad in 2019, in countries such as in Australia, United Kingdom (UK), Singapore, Europe, Canada, and Asia ( Berita, 2020). Taiwan is one of the most highly educationally developed economies in the world ( Guo, 2005, p. 134), and offers a lot of scholarships for undergraduate, master, doctor, double degree, and student exchange programs. Taiwanese universities are highly favored by researchers because of the quality education, competent teaching staff and the country is not too far from Indonesia so that more and more Indonesian students choose to continue their studies in Taiwan.

This study aims to investigate the experiences of the awardees of student exchange programs. In Indonesia, candidates for student exchanges with Taiwan are elected based on reciprocal partnerships set up by Memorandum of Understanding signed by Taiwanese and Indonesian universities. The student exchange offers free tuition for only two students as awardees. Those who join the student exchange program have to support themselves. The case presented in this research was the first time for the Indonesian university (IU) to have a reciprocal partnership with a Taiwanese university and to send their awardees abroad. This study focuses on the experiences of these two student exchange beneficiaries from Indonesia, who enrolled in the undergraduate program. This led to an investigation into the Indonesian exchange students' experiences at the Taiwanese university. In sum, this study attempts to answer the research question “What are the challenges and benefits of attending the student exchange program in Taiwan?”.

This study is focused on investigating students who come from developing countries and who do not have prior experience of learning in full English. This study presents various insights from the exchange students' experiences which will benefit future students studying abroad as student exchange awardees, particularly for future students who have no previous foreign study experience. It will also contribute to the education field of academicians, researchers, experts, or even the government, regarding the student exchange program, which affects aspects of life and study.

## Methods

### Participants and context

The participants in this study two college students, with the pseudonyms P1 and P2, consented to participate in this descriptive qualitative study. They were students in the Department of English Education at a private university in Indonesia. Participants were recruited after the researchers knew the information from the list of Indonesian student communities at the Taiwanese university to which they are going to. Besides, the researcher got information from their home university that both of the participants were chosen to be the first batch of student exchange ambassadors to represent their home university during the spring semester of 2020 (February to June 2020). Initially, they were elected as the first and second rank of hundreds of college students who took the student exchange written test and interview from the committee at their home university. However, despite their outstanding performances during the selection test from the committee, it was discovered that they did not have any experience regarding going abroad. This includes a lack of experiences of the flight travel system such as the process of check-in and finding the gates at the airport. It was the first time in their lives to apply for passports and visas, with this study's authors as volunteers, to guide them during preparation for departure and to welcome them in Taiwan. In conclusion, the criteria for the recruitment of study participants were: (1) admitted as exchange students; (2) voluntary participation; (3) coming from a rural area; (4) no experiences going abroad.

The exchange was to a national university in the east coast county of Taiwan. They officially registered for the student exchange program for one semester in an English department, and attended several courses such as fundamental Chinese, English writing, reading contemporary, western English literature, and critical approach to literature. Those courses were all taught in English. The students were used to studying in full Bahasa Indonesia in their home university, in the western part of Indonesia. The students' characteristics are shown in Table 1.

**Table 1.** Profile of participants.

Name	Age, years	Semester/year	Major	Student status	Religion
P1	20	6 ^th^/3 ^rd^	English Department	Undergraduate	Catholic
P2	20	6 ^th^/3 ^rd^	English Department	Undergraduate	Muslim

### Data collection

Data was gathered primarily through semi-structured interview questions ( Table 2) that were administered to the study participants after the completion of their exchange program. The purpose was to share their personal experiences in the Indonesian language for 30-60 minutes. The meeting was scheduled with participants and held in a location that was convenient. The audio from the meeting was recorded and field notes were made. Data were triangulated through an informal participant observation from P1 and P2.

**Table 2.** Semi-structured interview questions.

No	Questions
1	Would you please describe your personal information?
2	Would you please describe your experiences before leaving abroad?
3	How did you get elected as an exchange student representative?
4	What was your first impression of the flight abroad?
5	Would you please describe your first experiences at a Taiwanese university?
6	How was your first experience following the student exchange program?
7	What were new things you found in classroom activities?
8	How did you deal with the assignments during the exchange program?
9	What culture shock did you have?
10	How did you interact with the local and international students?
11	What difficulties did you face in completing the study as an exchange student?
12	What funny stories did you have from the beginning of your time in Taiwan?

For the interview, a set of open-ended questions was used to explore their experiences during the interview ( Hsieh & Shannon, 2005). Close-ended questions regarding age, religion, and other privacy questions were also collected. A WhatsApp group was created to allow for follow-up questions and responses to be sent between the researcher and participants. During the interview participants answered the questions without pressure.

Since the second author was a doctoral student at the same university with the participants, informal observations of participants were conducted by the researchers to see the participants' emotions during the exchange program. This was done by observing the two participants both on and off campus, for instance while going to the library, eating at the cafeteria, riding bicycles, window-shopping, or studying in the study room.

### Data analysis

The interviews were transcribed into English. Subsequently, themes for each interview were created to develop thematic grouping. A summary form of the interview was created and provided to the participants in order to obtain their feedback before the themes were finalized ( Creswell, 2013).

### Ethical considerations

This study was approved by Tarutung State Christian Religion Institute (Protocol number H.0206/F1000R/RSF/XII/2020). After due consultation, consent letters were provided by the home university and Taiwanese university.

Before the interview was conducted, the researchers sought participants' willingness by asking them to fill out and sign an informed consent sheet which explained the objectives of the study. Confidentiality was assured by using codes and pseudonyms prior to the interviews. After the data has been transcribed and analyzed qualitatively, the recorded data sheets were archived for confidentiality reasons.

## Findings

Challenges and Benefits are the two major themes in this research. Challenges include: (1) Departure and journey; (2) Difficulties in settling in; (3) Inability to use technology facilities. Benefits include: (1) Meeting new people and exploring new places; and (2) 21 ^st^-century classroom environments. These themes are discussed narratively in the following sections.

## Challenges

### Departure and journey

For the first time students were selected to be “student exchange program ambassadors” to represent their home university. Lecturers and staff were very excited because the university had succeeded in opening international doors, perhaps leading to an increase in the quality of their university through the international program. A week before the student's departure to Taiwan, the president of the university and staff gave an official “send-off party” at the home university. Motivational speeches were given, concluding with the tradition of giving the students tribal blankets, called “ *ulos*”, as a symbol of blessings. At the time of departure, everyone, including the students' family members, officially followed them to the airport in two of the university's official buses, a journey which took approximately three hours from the university to the airport. A printed banner on the bus explained that the students were being sent to Taiwan for student exchange programs.

*P1: “I felt emotional when a lot of people accompanied us to the airport and it also became a big spur for both of us to do our best at the Taiwanese university”.*

*P2: “My parents and family members came with me to the airport for the send-off and we received permission from my younger brother's school, so he could accompany us”.*

For the two students, it was the first time to fly by plane, so it was challenging for them to check in and transit at the airport. When they landed at Taiwan International Airport, they were confused about where to collect their luggage and ended up asking the airport employees for assistance. In addition, they did not have cash on them, so they tried to withdraw money at the airport's ATM machine, but this failed because their cards were not yet validated by their home bank for international transactions. This resulted in the students feeling sad and confused. They ended up borrowing money from an Indonesian student studying at the same campus with them. This student picked them up at the arrival hall, so they were able to buy food and milk to sustain them until they reached their destination, approximately four hours from the airport.

*P1: “We didn't eat for seven hours even though we had bread, we couldn't eat it because we felt dizzy from the flight”.*

*P2: “I did not know what to buy because it was my first time seeing the international food”.*

### Difficulties in settling in

Jenkins and Galloway (2009) investigated adjustment problems faced by international students studying in Taiwanese universities. Also Yi et al. (2003) found adjustment issues such as academic, physical health, financial, vocational, and personal/social issues in similar exchange programs.

The two students faced a lot of difficulties regarding understanding the local money because they did not change their Indonesian currency to Taiwanese currency when they were still in Indonesia. They thought that they would be able to use their ATM cards to get the Taiwanese currency, but unfortunately, their cards were not identified. This was reported to the local Indonesian local bank, but they were told that they would have to wait for a couple of days. This led to both students being depressed and miserable as they did not know if there would be somebody to lend them money until their cards could be used. This culminated in P1 crying at the convenience store near their dormitory because she did not want to borrow money. P2 called her parents to ask that they send money urgently through her senior's ATM and asked her senior to borrow money in the interim. Due to P1's situation, they discussed their difficulties on the phone with one of their lecturers in Indonesia, and their lecturer was able to contact a friend to ask them to lend the students enough money to get by.

Even though the two students belonged to different religions, they did not want to be separated from each other. Therefore, when P1 went to pray at church, P2 would accompany P1 to the church and wait for P1 in the lobby. When P2 went to pray at the Mushola, P1 would accompany P2 and wait. The other students asked P1 and P2 a lot of questions about Indonesia, for example, why Indonesian people like spicy food and how Indonesian people deal with their hot weather since it is a tropical country. Luckily, all the friends they met in Taiwan respected the religious backgrounds of both P1 and P2. For example, when they had to eat at a restaurant together with their international friends, everybody was really helpful to choose a vegetarian food for P2 since P2 is a Muslim and it is forbidden to eat pork. This friendly attitude is referred to as international relations, which means the ability of people from different countries to collaboratively work together to establish a good relationship ( Knight & Wit, 1997).

After living in Taiwan for one month, their home university asked P1 and P2 to take a photo in front of the logo of their university in Taiwan. This was done to make a big banner in front of their home university stating that they had officially studied at the Taiwanese university as student exchange program ambassadors. Three months later, the IU campus advised them to make a short video about their learning process, environment and others in order to provide an overview of the study process in the Taiwanese university. This was done so that their friends at IU could find out what their studies looked like. This was for promotional purposes to the public and at the same time, to inform the public that the IU is implementing the “goes international” campaign. This promotion can make both universities have a market branding and become highly reputable ( Sakamoto & Chapman, 2012). Also, this promotion can create good reputations for them that will lead to a competitive advantage ( Knight, 2004). After a couple of weeks, they became comfortable with many things in Taiwan that made them more interested in sharing their life experiences in their Taiwanese university.

### Inability to use technology facilities

At first, the students met with difficulties in getting water to drink at their dormitory, even though there were four dispensers in every corner of the floor; when they located them, they did not know how to use those dispensers, so they did not drink water that night. When they woke up the next morning, they had the bread that they brought from Indonesia for breakfast without drinking any water. They stayed hungry in the afternoon because they did not understand how to turn on the rice cooker to prepare lunch. After trying for four hours, P2 called their senior to teach them how to operate the electronic equipment in the dormitory area. Their first trial at cooking rice failed. Finally, they did not eat the rice, but ate more of the bread that they brought from Indonesia.

*P1: “We got confused and just felt awkward, and so we laughed at each other even though we were very hungry”.*

*P2: “We were so grateful when our senior came to visit us and he brought us some food and drinks, we might have fainted if he did not”.*

The two students were extremely pleased with the dormitory facilities and the condition of the rooms, which they described as comfortable, clean and safe. This made a great impression on the students, making them willing to stay for more than one semester.

*P1: “I was so sad when my room was separated from that of P2, but I still managed to be excited because I had a roommate who was a local student to be my new friend. Unfortunately, I do not feel totally comfortable with my roommate because she always turns on the air conditioner while sleeping, and I get cold every night. I went to the manager of the dorm to change my room, but he refused me”.*

*P2: “I do not have any specific problems with my roommate. I am a Muslim and have to pray five times every day, and she has no problem seeing me doing my habitual activities”.*

The students were surprised to see that every student had bicycles which were parked properly in the parking lot, and after a short stay, their senior gave them a bicycle each. For the first two weeks P1 rode with P2 because P1 was not good enough at riding a bicycle. So she had to practice for about two weeks until she was able to ride by herself.

The awardees felt too awkward to explore the university facilities themselves. So their senior invited them to have a campus tour. This included visiting the library, which the senior left the students to explore by themselves. However, the students did not go anywhere because they felt too awkward to go up to the next level of the floor, and instead they decided to sit and wait for the senior to come back and pick them up. In addition, the students did not know how to borrow books from the shelves or online through the library website. Their classmates showed them how to select and reserve books through the website. The library is usually open at 09:00 am to close at 22:00 pm, and most students come in the morning hours and stay until it is about to close. The library is very safe, meaning personal items can be left unattended.

*P1: “I saw a lot of students searching for books through the computers that are available on every floor. Many students copied their books by themselves using the photocopy machines that are available on every floor”.*

*P2: “There are a lot computers provided in our library, we only sign in with our student id number and we can use unlimited materials. We also get 100 pieces of paper for free to print for every semester. This library provides a small room for individuals to study, and a big room for a group to have a discussion”.*

Living in other countries, and exposure to other cultures generally creates goodwill and contributes to global peace and security ( Guruz, 2008). Therefore, since these student exchange awardees were able to adapt to their environment, it is very likely that the Indonesian government or the education sector will be interested in hiring these two students who have the problem-solving ability to make decisions independently ( Lai, 2018).

## Benefits

### Meeting new people and exploring new places

The cafeteria nearby the student's department provides a buffet, where food must be weighed before paying since payment is based on weight. This kind of payment system was very new for the students'. Even though hundreds of students come to this cafeteria, the students reported that everything was kept neat, clean and orderly.

*P1: “When I visited the buffet cafeteria alone, I was a bit nervous and I didn't know how to measure how much food I had already taken. Finally, after I weighed my food, it was super expensive, and I felt so worried about that I wish I had taken just a little food”.*

*P2: “Because I am a Muslim, I have to be careful to take own my food because beef and pork look alike on the stand”.*

Many restaurants in Taiwan open twice a day, including the restaurant near the student's college, which opens three hours per shift. The first shift is 11:00-14:00 pm, the second 17:00-20:00. Most of the local restaurants offer self-service, i.e. drinks, spoons, and chopsticks are gathered by the patrons and the patrons must clean their own tables. If people come late to the restaurant, they won't be served. All these rules are found in Taiwan and not in their home country Indonesia. The students reported that adjusting to local people's activities really helped them to be more disciplined and organized.

*P1: “I could not find mineral water in all the restaurants that I visited, but there was always black tea, jasmine tea and soup. I was surprised that most of the students eat in the restaurants every day instead of cooking at the dorm”.*

*P2: “I didn't expect Taiwanese food to be so bland. The menus had spicy written in them but for me, the meals were not spicy at all. I also discovered that most of the restaurants did not have a menu written in English. So I had to order orally from the waiters with gestures and showing them the food picture from my cell phone at the same time. What I really love buying from the restaurants are the delicious soft drinks”.*

Student exchange program awardees get the opportunity to make new friends with other international students and to also strike up good friendship with the local students ( Daly, 2007). They watch as many students do exercise at night even until mid-night and take showers afterwards. They enjoy buying fried chicken pie and a big cup of Boba milk tea at night. They go to sleep so late at night and wake up at 12:00 pm. Most of them skip their breakfast and eat lunch later. If the students had to come to the class in the morning, they brought their breakfast to the classroom. Also, they came to the class wearing clothers of their own choosing such as short pants, with sandals.

In addition to that, they have a bus that always comes based on their schedule. The bus is very convenient and the drivers are also friendly. The awardees used to say thank you afterwards and smiled when getting off the bus. The students pay for their rides by tapping their student identity card to the monitor close to the driver.

*P1: “Most of the passengers always say thank you and goodbye to the driver. We can only stop at the official bus stops. We cannot stop just anywhere we like and this is totally different from what happens in my own town”.*

*P2: “Trains and buses are widely used and the system is extremely well organized. We used an app for the bus schedule, and we used the website for the train schedule. It really helped us to navigate our transportation here”.*

They have an Indonesian students' community. At the beginning of the semester, this group of people got together in the auditorium to welcome the new students. They introduced themselves one after the other in front of their friends, revealing their personal profiles such as the name of their universities in Indonesia, their addresses, their majors and so forth. This is often the best time for them to make more friends and to learn more about Taiwan. However, due to the COIVD-19 pandemic, many field trips from the university were cancelled. Due to the welcome party cancelation, they found it difficult to get to know their fellow students from Indonesia. So their senior started inviting them to several activities outside the campus.

*P1: “We went to the river, lake and ocean together which was fantastic. We rode bicycles and explored the inside and the outside of the college together. In addition, I began to have more friends to go to church with every Sunday. The church was located far away from my dorm”.*

*P2: “There is always a pray day every Friday at the mosque located in the city, which I normally attend as a Muslim. It is about thirty minutes away from my dorm. After praying I used to get free Indonesian food for dinner, and I am so happy because it helps me to save money”.*

### 21 ^st^-century classroom environment

The lecturers that taught the students were Professors, and are experts in their various fields. Most of them graduated from outside Taiwan. These lecturers taught them in English. In the first week, they were a little confused by a Taiwanese lecturer whose English pronunciation was unclear. Their lecturers implored them to be active in class. For example, they participated in collaborative learning that encourages students to be creative to solve problems and develop a relationship with each other. Collaborating and working effectively with others make a lasting positive impact on individual student learning ( Saner et al., 1994). The lecturers applied 21 ^st^ century teaching methods in class. For example, they used a student centered method whereby students are used as facilitators to explain the materials to their peers through the learning by doing method. In the contemporary reading class, the student's reported that the lecturer brought stickers to class that were given to students who delivered questions, ideas and responses to the lecturer. Before the class ended, the lecturer would use the remaining ten minutes to give conclusions that helped the students to understand more about the class. In the critical approach to literature class, the students reported that the lecturer started the class with an e-learning system and followed-up with discussions that required the participation of about 4-5 students per group.

*P1: “I felt very nervous, scared, and not confident because I never had classmates who came from various countries before. Fortunately, all the lecturers and classmates were very cool and friendly so that I became more comfortable”.*

*P2: “Initially, I was not too shocked because there were a number of Indonesian students in the fundamental Chinese class. So I felt comfortable and not depressed. In another class, my Taiwanese friends were very friendly and helpful”.*

*P1: “Our department is very big, but we never got lost on our way to the classroom”.*

The students reported that they had good classmates. When they had assignments they has discussions in the library study room and café. When the semester was over, they had their own farewell party with their classmates. The students reported feeling very emotional at the end of the semester because they had to say goodbye to one another.

The classroom is one of the most important platforms for students to acquire knowledge and develop skills ( Kim et al., 2019). The students reported that their classroom facilities supported the learning process. These facilities included air conditioners, fans, projectors, comfortable seats, good lighting and other equipment, which made the environment very conducive to learning and this situation is totally than different at their home university. Therefore, all the activities in the classroom, including e-learning, really enhanced their 21 ^st^ century skills which includes critical thinking, communication, collaboration, and creativity ( Hadianto, 2019).

## Discussion

Living in other countries, and exposure to other cultures generally creates goodwill and contributes to global peace and security ( Guruz, 2008) even though there are a lot adjustment problems are faced in Taiwanese university ( Jenkins & Galloway, 2009) such as academic, physical health, financial, vocational, and personal/social issues in similar exchange programs ( Yi et al., 2003). The students reported that their classroom facilities supported the learning process. The classroom is one of the most important platforms for students to acquire knowledge acquisition and skills development ( Kim et al., 2019). These facilities included air conditioners, fans, projectors, comfortable seats, good lighting and other equipment, which made the learning environment to be very conducive and this situation is totally different at their home university. Therefore, all the activities in the classroom, including e-learning, really enhanced their 21 ^st^ century skills which includes critical thinking, communication, collaboration, and creativity ( Hadianto, 2019).

In addition, their lecturers implored them to be active in class, for example, to participate in collaborative learning that encourages students to be creative to solve problems and develop a relationship with each other; by collaborating and working effectively with others in order to make a lasting positive impact on individual student learning ( Saner et al., 1994), an example of positive impact is about the tolerance, as they belong to different nationalities, religion, race and so forth, they still respect each other and this kind of friendly attitude is referred to as international relations, which means the ability of people from different countries to collaboratively work together so as to establish a good relationship ( Knight & Wit, 1997). In addition, student exchange program awardees get the opportunity to make new friends with other international students and to also strike good friendship with the local students ( Daly, 2007). Therefore, since these student exchange awardees were able to adapt to their environment, it is very likely that the Indonesian government or the education sector will be interested in hiring these two students who have the problem-solving ability to make decisions independently ( Lai, 2018).

After living in Taiwan for one month, their home university asked P1 and P2 to take a photo in front of the logo of their university in Taiwan, so they could make a big banner in front of their home university stating that both of them had officially studied at the Taiwanese university as student exchange program ambassadors. Three months later, the IU campus advised them to make a short video about their learning process, environment and others in order to provide an overview of the study process in Taiwanese university so that their friends at IU could find out what their studies looked like. This was for promotional purposes to the public and at the same time, to inform the public that the IU is implementing the “goes international” campaign. This promotion can make both universities have a market branding and become highly reputable ( Sakamoto & Chapman, 2012), and this promotion could create good reputation for them that will lead to a competitive advantage ( Knight, 2004). After a couple of weeks, they became comfortable with many things in Taiwan that make them interested more than ever in sharing their life experiences in their Taiwanese university.

### Limitations and future research

Studies conducted to investigate the international student exchange programs have increased significantly in recent times. The present study did not investigate the student exchange program awardees' experiences in terms of motivation, cultural background, personality, and attitude toward living and studying as student exchange awardees at the Taiwanese university. Therefore, future research should attempt to include these and perhaps other variables that relate to the international student exchange programs at the Taiwanese university.

## Conclusion

This qualitative study depicts the activities of two student exchange program awardees who were allowed to study abroad in Taiwan. It also examines the obstacles they faced during their one semester at the Taiwanese university. According to these awardees, the experience was a sure pathway to expand their friendship network to various countries in the world and with people of different backgrounds and cultural aspects. The student exchange awardees had a chance to introduce Indonesian culture to the foreigners that they met, through activities that were both inside and outside of the university. They also had the opportunity to meet some people in the Indonesian community in Taiwan, which formulated a network to work and discuss together, and to exchange experiences and knowledge among themselves. Moreover, they got a chance to meet with an Indonesian individual who is married to a Taiwanese individual; and this helped them to partake in the Halal food meant for Muslims.

In addition, the students had the opportunity to travel to several cities around Taiwan. It made them feel different when compared to their home country in terms of culture, weather, lifestyle, people, and transportation. Although it was not easy at first for them to live in Taiwan as strangers, but as time went by, they ended up adjusting to a whole new lifestyle altogether, in an unfamiliar environment, and to speak a new language which comprises both English and Chinese. They succeeded in completing the mission given to them to study for one semester at a Taiwanese university. Once back in Indonesia, the students were uncertain how to adjust the student experience to their old lives back home and they felt new to their old group of friends. They discovered that they had grown as ambassadors, and acquired knowledge about the 21 ^st^-century learning environment. In conclusion, they became independent, appreciative of their country, culture understanding, and they became more open to new ideas, assertive, and more confident in themselves.

## Data availability

### Underlying data

Figshare: Raw Data Wolter, https://doi.org/10.6084/m9.figshare.14131646.v2 (Silalahi, 2021).

Data are available under the terms of the Creative Commons Zero “No rights reserved” data waiver (CC0 1.0 Public domain dedication).

## Consent

Written informed consent for publication of the anonymized interview transcript was obtained from the participants.
