# Supplementary material for: From frustration to enlightenment: experiences of student exchange program awardees in Taiwan
Source: F1000Res. 2021 Nov 29;10:468. Originally published 2021 Jun 14. [Version 2] doi: 10.12688/f1000research.51865.2 (PMC8637245; doi:10.12688/f1000research.51865.2)
Supplement: Peer review history

# Peer review: Reviewer response for version 2

**DOI:** 10.5256/f1000research.79666.r101433

**Published:** 2021-12-07

**Authors:** Gregory Siy Ching

**Affiliations:** 1 Research and Development Center for Physical Education Health and Information Technology, Graduate Institute of Educational Leadership & Development, Fu Jen Catholic University, New Taipei City, Taiwan

The recent revisions are acceptable.

Is the work clearly and accurately presented and does it cite the current literature?

Partly

If applicable, is the statistical analysis and its interpretation appropriate?

Not applicable

Are all the source data underlying the results available to ensure full reproducibility?

Yes

Is the study design appropriate and is the work technically sound?

Partly

Are the conclusions drawn adequately supported by the results?

Partly

Are sufficient details of methods and analysis provided to allow replication by others?

Partly

Reviewer Expertise:

NA

I confirm that I have read this submission and believe that I have an appropriate level of expertise to confirm that it is of an acceptable scientific standard.

# Peer review: Reviewer response for version 2

**DOI:** 10.5256/f1000research.79666.r101432

**Published:** 2021-12-01

**Authors:** Anna Riana Suryanti Tambunan

**Affiliations:** 1 Department Language Education and English Literature, Universitas Negeri Medan, Medan, Indonesia

No more comments.

Is the work clearly and accurately presented and does it cite the current literature?

Partly

If applicable, is the statistical analysis and its interpretation appropriate?

Yes

Are all the source data underlying the results available to ensure full reproducibility?

Yes

Is the study design appropriate and is the work technically sound?

Yes

Are the conclusions drawn adequately supported by the results?

Yes

Are sufficient details of methods and analysis provided to allow replication by others?

Yes

Reviewer Expertise:

TEFL, Intercultural Communication, English language and Literature, English Teaching and Learning.

I confirm that I have read this submission and believe that I have an appropriate level of expertise to confirm that it is of an acceptable scientific standard.

# Peer review: Reviewer response for version 2

**DOI:** 10.5256/f1000research.79666.r101434

**Published:** 2021-12-01

**Authors:** Xuesong (Andy) Gao

**Affiliations:** 1 School of Education, University of New South Wales, Sydney, NSW, Australia

This is a very small-scale inquiry but it has captured the voices of two Indonesian students in Taiwan (not much attention has been paid to these students). I am happy to see it in print.

Is the work clearly and accurately presented and does it cite the current literature?

Partly

If applicable, is the statistical analysis and its interpretation appropriate?

Not applicable

Are all the source data underlying the results available to ensure full reproducibility?

Yes

Is the study design appropriate and is the work technically sound?

Partly

Are the conclusions drawn adequately supported by the results?

Yes

Are sufficient details of methods and analysis provided to allow replication by others?

Yes

Reviewer Expertise:

Language education

I confirm that I have read this submission and believe that I have an appropriate level of expertise to confirm that it is of an acceptable scientific standard.

# Peer review: Reviewer response for version 1

**DOI:** 10.5256/f1000research.55075.r90099

**Published:** 2021-07-23

**Authors:** Gregory Siy Ching

**Affiliations:** 1 Research and Development Center for Physical Education Health and Information Technology, Graduate Institute of Educational Leadership & Development, Fu Jen Catholic University, New Taipei City, Taiwan

The current paper entitled "From frustration to enlightenment: experiences of student exchange program awardees in Taiwan" is an interesting study focusing on the study abroad experiences of two Indonesian students in Taiwan. However, as of the moment, the paper needs some reworking.

 The Abstract can be improved. Participants were not clear. At first, two students were recruited as ambassadors - this is misleading. It should directly say there were two participants in the study (why was the term 'ambassadors' used - as later repeated in the Methods section) and this should be expanded.

 The validity and reliability of the study might be affected since only two participants were interviewed. It would be hard to generalize the findings to all Indonesian students in Taiwan. However, the current study could be a starting point for a larger quantitative study, perhaps a survey generated from the current results.

 The literature review should be expanded. There are also some studies done using a qualitative lens that the authors were not able to review. For instance: A case study on the international students’ social adaptability in Taiwan: A qualitative study ****by Lewis *et al., *2012 ^1^. Although it's not that new, the authors can still review it.

 In general, the paper has potential but needs reworking.

Is the work clearly and accurately presented and does it cite the current literature?

Partly

If applicable, is the statistical analysis and its interpretation appropriate?

Not applicable

Are all the source data underlying the results available to ensure full reproducibility?

Yes

Is the study design appropriate and is the work technically sound?

Partly

Are the conclusions drawn adequately supported by the results?

Partly

Are sufficient details of methods and analysis provided to allow replication by others?

Partly

Reviewer Expertise:

Study abroad, internationalization of higher education, research teaching nexus, academic identity

I confirm that I have read this submission and believe that I have an appropriate level of expertise to confirm that it is of an acceptable scientific standard, however I have significant reservations, as outlined above.

## References

[1] : A case study on the international students’ social adaptability in Taiwan: A qualitative study. *International Journal of Research Studies in Psychology* .2012;2(1) : 10.5861/ijrsp.2012.161 10.5861/ijrsp.2012.161

# Response

**Published:** 2021-11-17

**Authors:** Wolter Parlindungan Silalahi

**Affiliations:** Institut Agama Kristen Negeri Tarutung, Indonesia

Dear Prof Gregory Siy Ching,

 We are so thankful for your generous suggestion for this study. We had revised this paper based on your suggestions, as follows:

1. The abstract: the writers had revised the participants' description instead of ambassador.
2. Since only two participants were interviewed that affects the validity and reliability, based on your suggestion we agree that it could be a starting point for a larger quantitative study.
3. The writer expanded the literature review in the introduction/background section.

Thank you~

# Peer review: Reviewer response for version 1

**DOI:** 10.5256/f1000research.55075.r88855

**Published:** 2021-07-22

**Authors:** Anna Riana Suryanti Tambunan

**Affiliations:** 1 Department Language Education and English Literature, Universitas Negeri Medan, Medan, Indonesia

This study is fascinating to publish. I learned a lot from this article about the exchange experiences of Indonesian students studying in Taiwan. However, there are some things to consider, especially in terms of the language presented in this manuscript which is sometimes unclear. For example, in the sentence: "Studying abroad is one of the most exciting experiences for a college student who is running a student exchange program, as it creates opportunities to learn, work, solve problems, and ensure a secured life," in the Abstract. In this sentence, the true meaning of studying abroad versus student exchange programs is not clear. The author may focus on only one program, for example, the student exchange program in Taiwan.

 In the Introduction section, it is also necessary to elaborate on the issues related to the student exchange program related to student frustration. It is recommended that the research questions appear in the last paragraph of the Introduction section.

 Then in the Methods section, a detailed explanation of how to recruit participants, collect data, and analyze data should be given.

 In the Discussion section, it is also necessary to add previous studies that support each finding. It would be better if it was elaborated according to the findings in the Discussion section.

 Finally, in the Conclusion section, there should be implications for this research.

 I hope my input will help the authors improve the article. Thank you.

Is the work clearly and accurately presented and does it cite the current literature?

Partly

If applicable, is the statistical analysis and its interpretation appropriate?

Yes

Are all the source data underlying the results available to ensure full reproducibility?

Yes

Is the study design appropriate and is the work technically sound?

Yes

Are the conclusions drawn adequately supported by the results?

Yes

Are sufficient details of methods and analysis provided to allow replication by others?

Yes

Reviewer Expertise:

TEFL, Intercultural Communication, English language and Literature, English Teaching and Learning.

I confirm that I have read this submission and believe that I have an appropriate level of expertise to confirm that it is of an acceptable scientific standard, however I have significant reservations, as outlined above.

# Response

**Published:** 2021-11-17

**Authors:** Wolter Parlindungan Silalahi

**Affiliations:** Institut Agama Kristen Negeri Tarutung, Indonesia

Dear Prof Anna Riana Suryanti Tambunan,

 Thanks for submitting your review, we have read your suggestions for our paper and revised our study based on your suggestions as follows:

1. The abstract: we have revised the election language presented in the manuscript.
2. The introduction: the writer has elaborated on the issues related to the student
  exchange program related to student frustration and proposed a research question of this study.
3. The methodology: the writers have revised the process of participants’ recruitment to participate in our study.
4. Discussion: we have added previous studies that support our findings. 
5. Conclusion section: we have revised the implications for this research.

Thank you Prof~

# Peer review: Reviewer response for version 1

**DOI:** 10.5256/f1000research.55075.r87499

**Published:** 2021-06-16

**Authors:** Xuesong (Andy) Gao

**Affiliations:** 1 School of Education, University of New South Wales, Sydney, NSW, Australia

The manuscript reports on a study that examined an underrepresented group of students in study abroad. It has some interesting insights for readers, but I do not think that the manuscript can be indexed in the current format. I suggest that the authors attend to the following issues before they resubmit their work for reconsideration.

1. First of all, the manuscript requires substantial rewriting to improve the quality of writing. Just take the Abstract as an example: I do not understand whether a study abroad student is someone who is running a student exchange program. I also do not understand how an exchange program will ensure a secured life for its participants. Should the authors report how data were collected before talking about how data were analyzed? Can they elaborate on the problems the two students faced before talking about their feelings? Are the challenges associated with the participants or experienced by them? There are so many similar writing issues throughout the manuscript, which discourages this reader from making a positive recommendation.
2. As mentioned earlier, the Introduction section has a lot of writing issues. I hope that the authors can improve the quality of writing and reorganize it so that it can have a better argument development. I also suggest that the authors focus on developing an argument for the study. What do we know about study abroad students? What do we need to know about study abroad students? Since the study involved two Indonesian students in study abroad in Taiwan, the authors should highlight that previous research has focused on students in developed countries or English medium universities (or students from developed countries to other countries). I think that the study could potentially address an important void. See Li, J. & Han, H. (2020) ^1^ and Song, Y. & Xia, J. (2020) ^2^.
3. The manuscript needs research questions (or a research question) that are clearly motivated by the Introduction section. I think that the interview schedule can be included as an Appendix but needs to be described in the main text. More details on data analysis are needed. What did the authors do to enhance the quality of the analysis?
4. The results should be presented as answers to the research question. I believe that the authors should be more selective in choosing themes for inclusion. It is better to organize the presentation of findings into two groups: challenges and benefits. It is also important to develop sub-themes under the two major categories (challenges and benefits). For instance, under the category of challenges, the authors can present linguistic challenges, cultural challenges or social challenges, etc. Otherwise, I just found that the themes in the findings are not always relevant, for instance, departure and journey. We need findings that constitute significant insights into the two Indonesian students' experiences. The current findings as presented are highly descriptive and not sufficiently engaging.
5. I suggest that the authors include a Discussion section or add some discussion to highlight in what sense the findings constitute important knowledge contributions to the field. It is not clear to this reader in what sense the study advances our understanding of study abroad students. Limitations should be presented before the Conclusion and can be presented as the directions for further research.

I do hope the authors seriously consider my comments for revision. All the best wishes.

Is the work clearly and accurately presented and does it cite the current literature?

Partly

If applicable, is the statistical analysis and its interpretation appropriate?

Not applicable

Are all the source data underlying the results available to ensure full reproducibility?

Yes

Is the study design appropriate and is the work technically sound?

Partly

Are the conclusions drawn adequately supported by the results?

Yes

Are sufficient details of methods and analysis provided to allow replication by others?

Yes

Reviewer Expertise:

Language education

I confirm that I have read this submission and believe that I have an appropriate level of expertise to confirm that it is of an acceptable scientific standard, however I have significant reservations, as outlined above.

## References

[1] : Learning to orient toward Myanmar: ethnic Chinese students from Myanmar at a university in China. *Language, Culture and Curriculum* .2020; 10.1080/07908318.2020.1858095 1-19 10.1080/07908318.2020.1858095

[2] : Scale making in intercultural communication: experiences of international students in Chinese universities. *Language, Culture and Curriculum* .2020; 10.1080/07908318.2020.1857392 1-19 10.1080/07908318.2020.1857392

# Response

**Published:** 2021-11-17

**Authors:** Wolter Parlindungan Silalahi

**Affiliations:** Institut Agama Kristen Negeri Tarutung, Indonesia

Dear Prof Xuesong (Andy) Gao,

 We thank you most warmly for your suggestions to improve the quality of the writing. The writers have revised the paper regarding your suggestions as a reviewer as follows:

1. In the background part of the abstract, the writers have organized the idea of the sentences based on your suggestions, from "Studying abroad is one of the most exciting experiences for a college student who is running a student exchange program, as it creates opportunities to learn, work, solve problems, and ensure a secured life", to "This study investigates two Indonesian students who participated in a semester at a Taiwanese university. It aims to explore the Indonesian student exchange program awardees’ experiences by examining both the obstacles and benefits of attending the Taiwanese university."
2. The introduction: we have revised the research question and the description of the participants' criteria. Previously, we didn't write these two points.
3. The result: we have organized the themes and sub-theme based on your suggestions.
4. Previously, we put the result and discussion in the same part. You suggested we separate them into different sections and we had revised it.
5. We have put the "limitations and future research" before the conclusion as suggestions as follows "Limitations should be presented before the Conclusion and can be presented as the directions for further research".
